# Feasibility of Peritoneal Dialysis in Extremely Low Birth Weight Infants

**Published:** 2012-10-01

**Authors:** Francesco Macchini, Agnese De Carli, Sara Testa, Rossella Arnoldi, Stefano Ghirardello, Gianluigi Ardissino, Fabio Mosca, Maurizio Torricelli, Ernesto Leva

**Affiliations:** Department of Pediatric Surgery, Fondazione IRCCS Cá Granda, Ospedale Maggiore Policlinico, Milan, Italy.; 1 Neonatal Intensive Care Unit, Fondazione IRCCS Cá Granda, Ospedale Maggiore Policlinico, Milan, Italy.; 2 Pediatric Nephrology and Dialysis Unit, Fondazione IRCCS Cá Granda, Ospedale Maggiore Policlinico, Milan, Italy.

**Keywords:** Extremely low birth weight infants, Peritoneal dialysis, Acute renal failure

## Abstract

Acute renal injury is common in extremely low birth weight (ELBW) infants with a frequency ranging from 8% to 24%. Peritoneal dialysis (PD) has been used only occasionally in ELBW. We report our experience and share the solutions used to tackle the difficulties rising from the small size of this type of patients. PD was successfully performed in three ELBW infants with acute renal failure. A neonatal, single-cuff, straight Tenckhoff catheter was placed in 2 patients, while a Broviac single cuff vascular catheter was used in another. PD was feasible and effective in all children. Leakage was observed with Tenckhoff catheters, but this did not impair the PD efficacy. The technical difficulties were related to the size and shape of the peritoneal catheters, not easily fitting with the very thin abdominal wall of the preterm infants. We conclude that PD is feasible and effective, can be considered as the rescue therapy in preterm ELBW infants with acute renal failure.

## INTRODUCTION

The frequency of acute renal failure (ARF) in extremely low birth weight (ELBW) infants ranges from 8% to 24% (1-4). Although peritoneal dialysis (PD) is considered the treatment of choice for most newborns and older infants with renal failure, it has been used only occasionally in ELBW infants. Critically ill neonates are at risk for acute kidney injury (AKI) as they have frequent infections and are exposed to nephrotoxic drugs (1, 4). Indications for dialysis in the newborn are based on evidence of fluid overload (>15%) or hyperkaliemia (> 7 mEq/l) or severe hyperazotemia (BUN >100 mg/dl) if unresponsive to treatment. So far, premature newborns, especially ELBW infants, have not been considered eligible for PD because of technical limitations (unavailability of small sized catheters and volume cyclers) as well as high infection rate. While PD has been reported in VLBW infants, only recently this technique has been described in ELBW (5-8). In the present work, we report our experience on PD in 3 ELBW infants with acute renal failure.

## CASE SERIES

Case 1:

 An in-born 700g twin male, product of a pregnancy with diamniotic-dichorionic placentas was delivered by premature cesarean section because of placental abruption. The other twin died prenatally. The child had Apgar scores of 2, 8 and 8 at 1, 5 and 10 minutes, respectively, and required high-frequency oscillatory ventilation for severe respiratory distress. A patent ductus arteriosus (PDA) was surgically treated at 12th day of life, after failure of medical therapy (ibuprofen and indomethacin). Multiple haemotransfusions were required because of severe anemia. US scan ruled out renal malformations. Following anti-prostaglandin treatment of PDA, a progressive deterioration of renal function, poorly responsive to conservative treatment, was observed and renal replacement treatment was started with intermittent hemofiltration (2 sessions). Due to persistent anuria, PD was started (maximum s-creatinine 5.86 mg/dL; urea 242 mg/dL; s-K 3.95 mEq/L). A neonatal, straight, single-cuff Tenckhoff catheter was placed with a paramedian entry-site, without any subcutaneous tunnel. PD was started 24 hrs after implantation and was continued for 3 days, with a fill volume ranging from 10 to 30 mL/kg and 45 minutes of dwell time. 

Minimal leakage of peritoneal fluid was observed from the exit site without impairment of PD efficacy; no other catheter-related complications were observed. Diuresis remerged 3 days after PD had been started and the catheter was removed 9 days after (Table 1).

**Figure:**
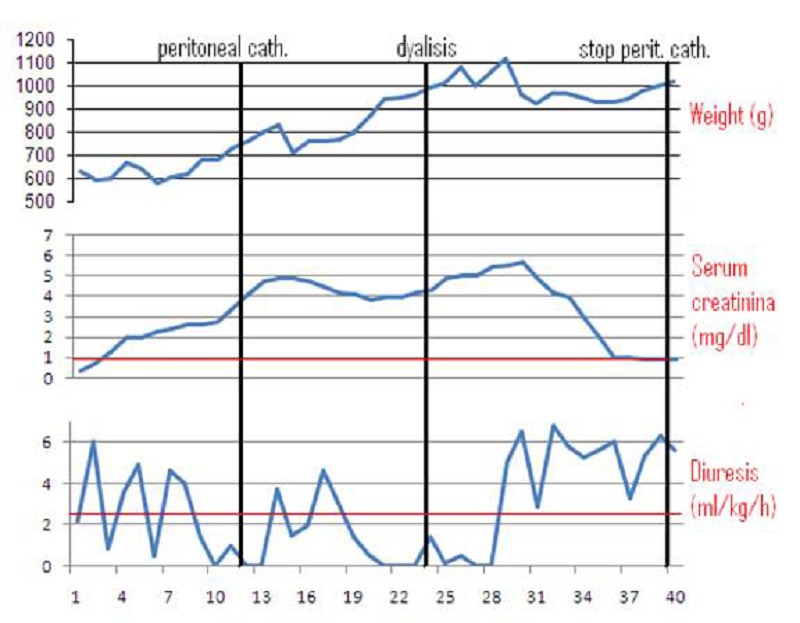
Table 1

Case 2:

 A 630g in-born male, with an antenatal diagnosis of oligohydramnios, left multicystic kidney and right hydronephrosis was born at 28 weeks of gestational age. After the cesarean section the child presented an Apgar score of 5, 8 and 8 at 1, 3 and 5 minutes, respectively. Shortly after delivery, the newborn was addressed to high-frequency oscillatory ventilation. The occurrence of sepsis required multiple haemotransfusions and antibiotic therapy. Following the infectious complication and the treatment with Ceftazidime, a rapid deterioration of the renal function was observed (maximum s-creatinine 4.7 mg/dl, urea 96 mg/dL, diuresis 0.5 ml/kg/day). Due to fluid overload (body weight of 830g on day 12) and persistent oliguria, unresponsive to diuretics, on the 10th day of life the infant became anuric, so that the decision of positioning a peritoneal catheter for dialysis was taken. A neonatal 13 FG Tenckhoff, straight, single-cuff catheter was implanted with a paramedian entry-site. The poorly represented peritoneal layer and the large caliber of the catheter and of the internal cuff did not allow a firm fixation of the catheter to the abdominal wall; therefore fibrin glue was applied on the external layer of the peritoneal membrane, as suggested by previous reports [9]. 

PD was started 8 days after surgery, with small fill volume (10 ml/kg) and a dwell time of 45 minutes, gradually increased to 20 ml/kg. Only partial exchanges were performed due to the occurrence of leakage. No other catheter-related complications were observed.

A progressive restoration of diuresis and of renal function (serum creatinine 1 mg/dl, diuresis 4 ml/kg/day) was obtained, so that PD was discontinued and the catheter removed after 27 days (Table 2). 

**Figure:**
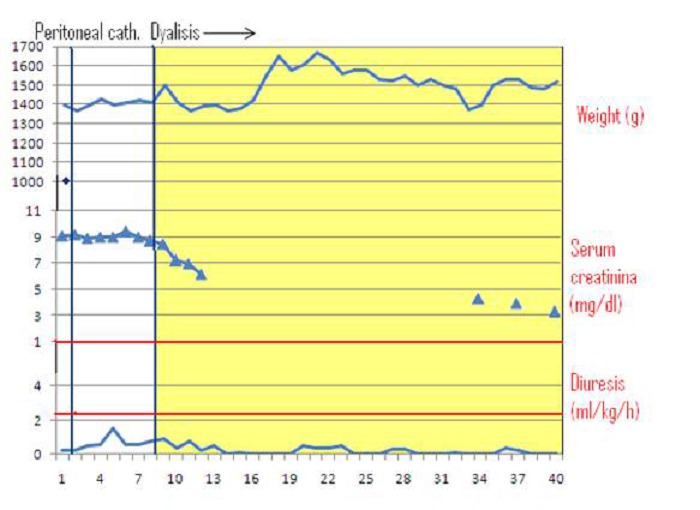
Table 2

A small laparotomy was required because of multiple adhesions between the cuff and the abdominal wall. Cystourethrography excluded vesicouretheral reflux and US-scan showed the reduction of the right hydronephrosis. At a 2 years follow-up, the renal function was normal.

Case 3:

 An out-born 1.097-g male, product of a monozygotic twin gestation complicated by twin-to-twin transfusion was referred to our Unit at the 19th day of life for oliguria, peripheral edema, massive weight gain (1.397-g) and congestive heart failure. 

The prenatal course was complicated by significant oligohydroamnios and growth retardation. He was delivered at the 30 weeks gestation because of worsening of the cardiac function. The APGAR was 8 both at 1 and 5 minutes. An ultrasound (US) examination performed at birth, showed a mild bilateral renal hypoplasia. Immediately after admission in our NICU, he was intubated and high-frequency oscillatory ventilation was started. A peritoneal catheter was implanted and PD started 5 days later. A Broviac, single Dacron cuff, 6.6 Fr vascular catheter was chosen, both for its reduced caliber and its higher flexibility as compared to Tenckhoff catheters previously used. A curved tunnel course (swan-neck) with the exit-site on the contralateral abdominal region was easily performed. PD was successfully carried out, without any complication; fill volume was progressively increased from 10 to 40 ml/kg.

A rapid improvement of clinical conditions and of chemistry was observed (Table 3). Due to the severe renal parenchymal damage no recovery of renal function was observed and home PD was continued.

**Figure:**
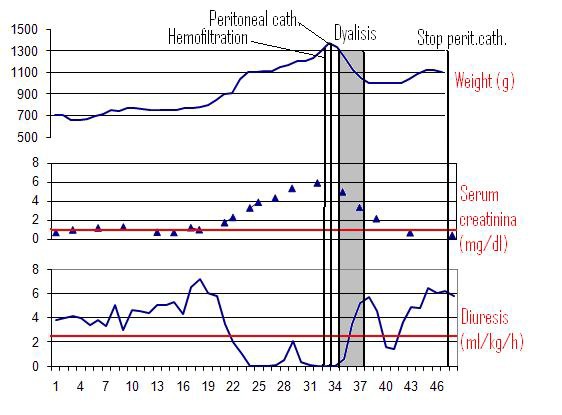
Table 3

## DISCUSSION

As reported in the literature, the main causes of renal failure in preterm infants are drugs toxicity, hemodynamic disturbances and sepsis (1-4, 6). Recent reports suggest that prematurity by itself may represent an independent risk factor for renal failure due to incomplete nephrogenesis and immature vaso-regulation (10, 11).

In the neonate, when conservative management fails, effective treatments are represented by continuous hemofiltration and PD. Preliminary and anecdotic reports suggest that PD has the potential to lead to better results in VLBW infants in case of acute kidney injury. Kanarek et al (3) described a successful case of 30 hours-PD in a VLBW infant with metabolic acidosis and hyperkalemia. Sizun et al (7) reported 3 cases of PD in VLBW infants. A retrospective review of PD in newborns demonstrated the efficacy of PD in the first 60 days of life, with a mortality rate in infants &< 1 year undergoing PD between 10 and 35 % (12). Two recent studies reported the experience of PD in VLBW infants (5, 6). PD seems to have a theoretical advantage in the premature neonates due to the large peritoneal surface area to body ratio with a consequent improvement in dialysis efficacy (4). Reported PD complications include peritonitis, exit-site and tunnel infection, catheter obstruction, abdominal wall hernia and bowel perforation secondary to erosion from the catheter (2, 7, 13-15).

In the present experience, 3 peritoneal catheters were implanted in 3 ELBW infants with acute renal failure. A neonatal, 13 FG Tenckhoff, straight, single cuff catheter was used in 2 patients, while a Broviac, 6.6 FG, single cuff, double lumen catheter was positioned in the last one. In all cases PD was performed with peritoneal fluid medicated with antibiotics and heparin. Dialysis was feasible and effective in all the children. Leakage was observed only in the 2 patients with Tenckhoff catheter, though it did not significantly affect the efficacy of PD. Whenever possible, the occurrence of leakage can be reduced by postponing the beginning of peritoneal dialysis, for some days after implantation of the catheter.

The present experience, although in a limited number of patients, suggests the following technical points:

a) Surgical placement of the Tenckhoff catheter in ELBW is challenging, due to the discrepancy between catheter size and abdominal wall thickness; the tissue repairing process in these infants is slow due to the cathabolic conditions.

b) An adequate subcutaneous tunnel in ELBW infants is difficult to obtain using a Tenckhoff catheter, because of its large caliber: the risk of infectious complications and leakage is increased.

c) Fibrin glue in preventing leakage is useful but it may complicate catheter removal, as reported in case 2.

As reported by a recent study (8), the use of a vascular catheter, with lower caliber, higher flexibility and small Dacron cuff, overcomes the above mentioned difficulties, allowing an easier and safer implantation. The incidence of infective complications can be reduced using medicated peritoneal fluids.

## CONCLUSION

Taken into account that there are no available data on the feasibility and efficacy of haemodialysis, PD can be considered the only rescue therapy in ELBW infants with acute renal failure. Peritoneal dialysis in the ELBW infants appears a feasible technique, without major complications. However, technical difficulties suggest that more suitable catheters for preterm infants are needed.

## Footnotes

**Source of Support:** Nil

**Conflict of Interest:** None declared
